# A mini-review on the impact of common gorse in its introduced ranges

**DOI:** 10.1007/s42965-022-00239-9

**Published:** 2022-05-02

**Authors:** Hansani S. S. Daluwatta Galappaththi, W. A. Priyanka P. de Silva, Andrea Clavijo Mccormick

**Affiliations:** 1 Department of Zoology, Faculty of Science, University of Peradeniya, Peradeniya, Sri Lanka; 2 School of Agriculture and Environment, College of Sciences, Massey University, Palmerston North, New Zealand

**Keywords:** Biological invasion, Detrimental effects, Exotic plants, Native ecosystems, Potential benefits, *Ulex europaeus*

## Abstract

It is indisputable that invasive plant species strongly impact the ecosystems they invade. Many of such impacts can be negative and threaten the local species through competition, environmental change, or habitat loss. However, introduced plants may also have positive roles in the ecosystems they invade. This review extracted information from reports on common gorse (*Ulex europaeus*), one of the top 100 invasive plants on the earth, including its detrimental effects and potential beneficial roles in invaded ecosystems. The reduction of native fauna and flora are the main harmful effects of common gorse identified by the literature review. Soil impoverishment and fire hazards are other negative impacts reported for common gorse that could affect agricultural systems and local economies. Despite the negative impacts, reports of positive ecological services provided by common gorse also exist, e.g., as a nursery plant or habitat for endangered native animals. We also reviewed the known human uses of this plant that could support management strategies through harvest and benefit the local communities, including its use as biofuel, raw matter for xylan extraction, medicine, and food. Finally, our review identified the gaps in the literature regarding the understanding of the beneficial role of common gorse on native ecosystems and potential human uses, especially in the tropics.

## Biological invasion and implications

Biological invasion is considered as one of the major environmental challenges worldwide (Sala et al. 2000; Pejchar and Mooney 2009; Vilà et al. 2010; Simberloff et al. 2013; Schirmel et al. 2016). Scientists have tried to define biological invasion in various ways. Van der Velde et al. (2006) described the biological invasion as the expansion of species' ranges into new areas. According to Simberloff (2013), biological invasion is the introduction and establishment of species into novel geographical ranges where they are capable of proliferating and spreading rapidly.

Undoubtedly, biological invasions have considerable impacts on the local ecosystems. In some cases, invasive species threaten native species directly through predation, competition, or parasitism. For example, native mammal communities in Australia have been threatened by predation of invasive feral cats and red foxes (Doherty et al. 2015; Woinarski et al. 2015). In other cases, hybridization leads to the extinction and loss of native genotypes. The genetic integrity of native California tiger salamander (*Ambystoma californiense*) have been threatened due to the invasive tiger salamander (*A. tigrinum*) while the genetic diversity of California cordgrass (*Spartina foliosa*) is affected due to the invasive Atlantic smooth cordgrass (*S. alterniflora*) (Ayres et al. 1999, 2004; Riley et al. 2003; Strong and Ayres 2013). Indirect impacts are also common due to habitat modification, changes in biotic interactions, and alteration of ecosystem processes (Pimentel et al. 2001; Simberloff et al. 2013; Mačić et al. 2018; Atlan and Udo 2019; Bartz and Kowarik 2019). However, invasive species can also be used by native species and humans to their advantage. While negative impacts are often reported and have been inextricably linked with alien and invasive species (Guerin et al. 2018), there is evidence suggesting that the presence of some invasive species can have positive impacts on the native communities that co-exist with them. For instance, frugivorous birds in Kenya are benefiting from the invasive exotic guava plants (*Psidium guajava*), which has become a preferred food source for native species (Berens et al. 2008). Invasive Australian *Acacia* spp. are popular among native communities in South Africa and Madagascar as fuel plants, showing that invasive plants can also be used by some local human populations to their advantage (de Neergaard et al. 2005; Kull et al. 2007).

Schlaepfer et al. (2011) highlighted the conservation value of non-native species, as these are more likely to persist in time than native species and provide ecosystem services under rapid environmental change scenarios. Invasive plants can provide shelter, reproductive or nesting sites, and alternative food sources for detritivores, pollinators, herbivores, and predators (Bowers et al. 1992; Nagy et al. 1998; Longcore 2003; Wonham et al. 2005; Levin et al. 2006; Effah et al. 2020). Some invasive species can also modify soil properties benefiting other plants and soil biota (Ehrenfeld 2003; Lee et al. 2012; Tun et al. 2020). Invasive *Pinus contorta* have been proven to enrich the soil with lignin, P, Mg, and Mn (Ågren and Knecht 2001; Ehrenfeld 2003). Beyond the ecological aspects, some alien invasive species have cultural or economic importance to local communities providing food, medicine, fuel, or fodder. For example, in Jorhat (India), an invasive plant, *Alternanthera tenella* is used as a vegetable and has a medicinal value, while another invasive plant, *Chamaesyce hirta,* has both medicinal (against anemia, asthma, and bronchitis) and insecticidal (controlling aquatic pests) properties (Das and Duarah 2013). However, the positive influence of invasive alien species on native biodiversity and local human populations is still poorly understood (Hanley and Roberts 2019).

Severns and Warren (2008) pointed out the importance of selectively controlling invasive exotic plants which may provide habitats for native and endangered species. For instance, *Euphydryas editha*, an endangered butterfly species in the Pacific Northwest of North America, switched from an unknown native larval host plant to an exotic host (*Plantago lanceolata*). The endemic Australian bird *Malurus cyaneus* (Superb Fairywrens) has higher nesting success in areas invaded by Blackberries *Rubus fruticosus* L. compared to native vegetation in Armidale, Australia (Nias 1986). Pearson (2009) described that invasion of western North American grasslands by the perennial forb *Centaurea maculosa* provides webbing surfaces, which ultimately increase native spider densities. Effah et al. (2020) found a high arthropod diversity and abundance associated with Scotch broom (*Cytisus scoparius*) in the Central Plateau of New Zealand, suggesting that native arthropods exploit additional resources provided by this invasive plant. Given the extent of expansion and the impossibility to eradicate or control many invasive plants (Head et al. 2015; Souza-Alonso et al. 2017), it is essential to identify and incorporate the beneficial impacts of these species in policy and management frameworks, which are primarily focused on negative impacts (Vimercati et al. 2020). *Ulex europaeus* L. (common gorse; USDA Plants Database 2021), is a widespread invasive species known to affect native fauna and flora in its invasive range. Though this plant species has been identified as an alien invasive species in diverse ecological settings, comparative evaluations have not been done to understand the negative and positive impact of invasiveness of this plant. In this review, we will explore reports on the invasive plant *U. europaeus* L., biology and invasiveness of gorse, its harmful effects, and potentially beneficial roles in invaded ecosystems as well as human uses.

## Common gorse biology and invasiveness

Common gorse is a heliophile evergreen shrub, also known as European gorse, furze, or whin (León Cordero et al. 2016; Andreas et al. 2017; ISSG 2020). The plant usually grows up to 1 – 4 m from its woody multi-branched root system. Leaves are three-parted in young plants and are reduced to scales or modified into thick spines when mature. Flowers are five-petal-yellowish and hairy seedpods that grow up to 2 cm long (Andreas et al. 2017, Figs. 1 and 2). *Ulex europaeus* belongs to the Family Fabaceae (Leguminosae) that can fix atmospheric nitrogen via rhizobial symbionts, causing changes to the soils in which they grow (Andreas et al. 2017; Sabagh et al. 2020). The native ranges of common gorse are Western Europe, primarily the Atlantic coast of Europe (the British Isles including Ireland) and northwest Africa (Hill et al. 2008; Magesan et al. 2012; Andreas et al. 2017). However, it is currently distributed in more than 50 countries around the world including the United States of America, Canada, South American countries (e.g., Colombia, Chile and Uruguay), Middle East, New Zealand, Australia,   remote islands such as Mauritius, Saint Helena, and some Asian countries (India and Sri Lanka) (Hill et al. 2000, 2008; Marambe 2001; Leary et al. 2006; Altamirano et al. 2016; Kariyawasam and Ratnayake 2019b; ISSG 2021).

**Fig. 1:**
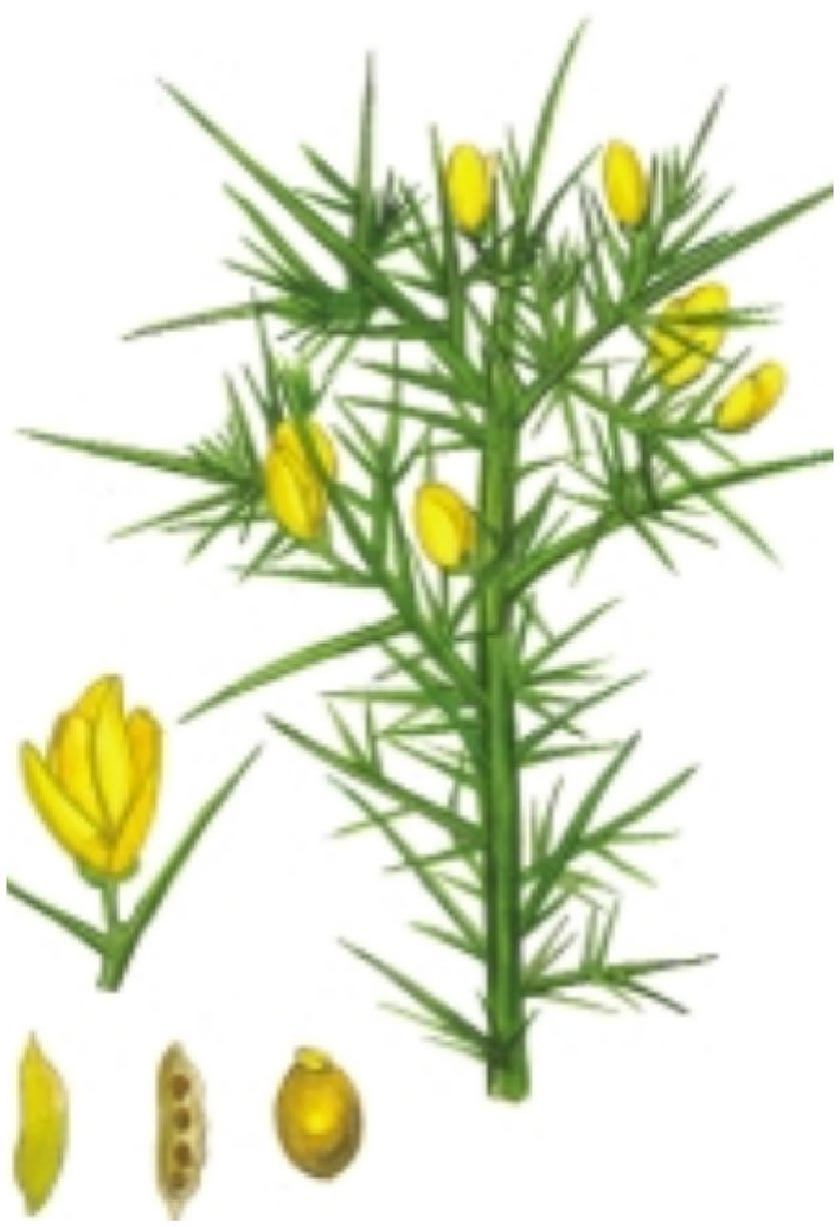
Habit sketch of common gorse plant (GISP 2005)

**Fig. 2:**
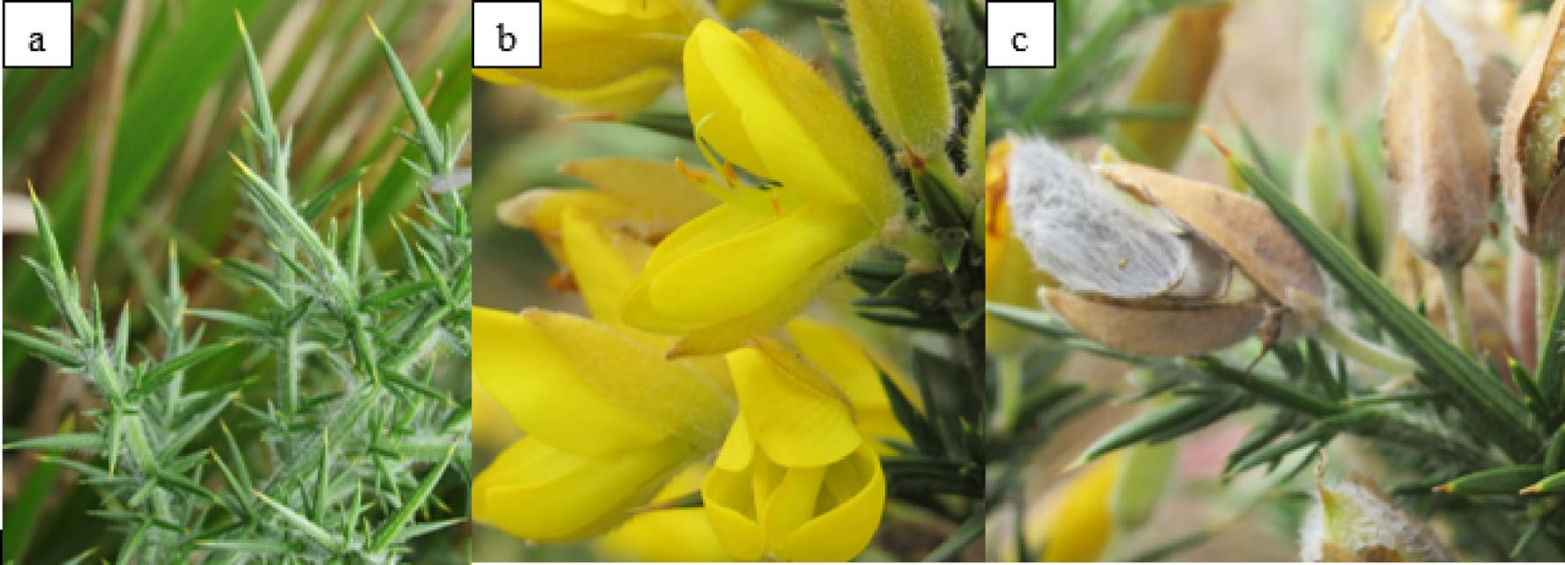
Key traits of common gorse. **a**. Spines **b**. Flowers **c**. Seed pods (PC: Hansani S. S. Daluwatta Galappaththi)

This plant was deliberately introduced to most of these countries as an ornamental plant for fencing, erosion control, and fodder for livestock (Lee et al. 1986; Andreas et al. 2017; Atlan and Udo 2019; Broadfield and McHenry 2019). However, the plant has rapidly spread in the introduced areas due to its life-history traits such as prolific flowering and seed production, high tolerance to a wide range of temperatures, increased ability to fix nitrogen, and large evolutionary potential which facilitates its competitive success (Atlan et al. 2015a; Broadfield and McHenry 2019). Further, water and fire-resistant hard seeds of this plant are viable in the soil for more than 50 years (Sullivan and Hutchison 2010). The production of such high-quality seeds allows this plant to grow rapidly and spread widely (Richardson et al. 1996). Plant invasion has traditionally been associated with negative effects on the diversity, abundance, and structure of native plants and animal communities (Levine et al. 2003). In such cases, management practices are often implemented to minimize the negative impact of common gorse on landscapes (Broadfield and McHenry 2019). Mechanical removal of mature plants and/or juveniles and land clearing, burning, applying herbicides, and biological control are some of the strategies used around the world to reduce the adverse effects of common gorse (Hill et al. 2008; Broadfield and McHenry 2019). There is an ample body of literature examining the control strategies aligned with the negative impacts, especially targeting the suitability of biological control in invaded geographical ranges (Chater 1931; Clements et al. 2001; Andreas et al. 2017; Atlan and Udo 2019; Broadfield and McHenry 2019). However, in many cases, due to the extension of the invasion, eradication is no longer possible and management can be costly or ineffective (Krause et al. 1988; Barker 2008; Mbatha 2016). In these scenarios, it is worthwhile to develop a different perspective to explore potential ecological benefits and human uses associated with *U. europaeus*.

## Detrimental effects of gorse to invaded ecosystems

The impact of common gorse in invaded ecosystems threatens native biodiversity and affects the soil quality and composition and thereby the agriculture, economy, and environmental health. These detrimental effects are discussed with examples below.

### Threats to the native ecosystems

The rapid infestation of common gorse has detrimental impacts on natural habitats (Egunjobi 1969; Zabkiewica 1984; Hill and Sandrey 1984, 1986; Richardson et al. 1996; Gouldthorpe 2009; Roberts and Florentine 2021). For instance, ISSG (2021) documented that the native plants at the red-listed Canadian Garry Oak Ecosystems have been displaced by the gorse invasion. Open grasslands are under the threat of gorse infestation as they convert the grasslands into thorny shrublands (ISSG 2010). Cordero et al. (2016) reported that common gorse has stimulated the colonization of woody species and thereby altered the forest-grass cover in forest-grassland mosaics of southern Brazil. Common gorse produces 200 kg ha^–1^ of Nitrogen annually during the rapid dry-matter accumulation period. This increased nitrogen level of the soil promotes the growth of other weedy species (Soto and Diaz-Fierros 1997; Drake 2011; Magesan et al. 2012; ISSG 2021). The dense, spiny thickets of common gorse influence the diversity and life-history traits of native plants, inhibiting the growth of native vegetation (Grubb et al. 1969; Lee et al. 1986; ﻿ Cordero et al. 2016) and affecting the subsequent succession process (Bellingham et al. 2005; Sullivan et al. 2007). In New Zealand, the *Ulex* thickets negatively affect plantations and decrease forest growth and development while competing with other plant species for water and nutrients (Richardson et al. 1996; Magesan et al. 2012). According to Harris et al. (2004) native mānuka (*Leptospermum scoparium*) and kānuka (*Kunzea ericoides*) scrub communities in New Zealand have been replaced by the gorse dominancy. National parks, forest reserves, riparian habitats, and bushland margins are severely affected by common gorse infestation (Gouldthorpe 2009; ISSG 2010). For instance, by 2004, 4000 ha of Humuula pastureland in Mauna Kea on the island of Hawaii were infested by gorse. The endangered flora and fauna in the protected Hakalau Forest National Wildlife Refuge, Hawaii were negatively affected due to the common gorse infestation (Leary et al. 2006). Studies have reported the significant effect of gorse on the biodiversity of temperate island ecosystems such as the Gulf Islands and Vancouver Island in Canada, and Tasmania in Australia (ISSG 2010). For instance, *Acacia axillaris* (midlands wattle), *Callitris oblonga* (South Esk pine), *Epacris apsleyensis* (Apsley heath), *Prasophyllum tunbridgense* (Tunbridge leaforchid), *Stonesiella selaginoides* (clubmoss bushpea), *Spyridium lawrencei* (small-leaf Spyridium), *Hibbertia basaltica*, *Bertya tasmaniaca* and *Pterostylis ziegeleri* are severely affected plant species in Tasmania by gorse infestation (Gouldthorpe 2009). There are, however, fewer studies investigating the effect of gorse invasion on tropical countries, where resources for management are often limited and invasion rate is high. 

### Impacts on the soil and the water

Cumberland (1944) described gorse as an effective soil stabilizer to control soil erosion. This particular use prompted its deliberate introduction into new ranges (Andreas et al. 2017). As a Leguminosae plant, gorse has the ability to fix nitrogen. While this could be advantageous in nutrient-poor soils, on healthy soils it would promote the leaching of excess nitrogen that would increase the soil acidity (Grubb and Suter 1971; ISSG 2010). The acidified soil alters and modifies the nutrient regimes affecting the native vegetation (ISSG 2010). The intensified nitrogen amount in the soil could indirectly affect the water quality of the ecosystem overstimulating the growth of algae and aquatic plants leading to eutrophication (Smith et al. 1999). As a result, the water bodies get clogged and the dissolved oxygen is reduced. Ultimately, this would suppress the aquatic flora and fauna diversity (Khatri and Tyagi 2015; FAO 2017; USGS 2021). Stewart et al. (2019) recently reported that common gorse-dominated catchments have higher nitrate concentrations. According to Mason et al. (2016), the common gorse cover of Ruamahanga catchment, New Zealand was 596 ha and the model-based leaching estimation showed that gorse accounts for 1.7% – 2.4% of total Nitrogen leaching in this catchment. This is equivalent to leaching from 1200 to 1800 ha of pasture across both dry stock and dairy land uses. With over 5000 ha of common gorse cover, estimated *N* leaching from gorse accounts for 12% – 25% of the catchment, and the expected increase in N leaching is equivalent to leaching for 9000 – 14,000 ha of pasture. Further, the altered nutrient regime changes the soil microclimatic conditions favouring other invasive weeds (ISSG 2010). Common gorse weeds also have a high capacity to absorb soil nutrients such as calcium, magnesium, sodium which affect the soil cation balance and thereby the soil health (Zabkiewicz 1976; MacCarter and Gaynor 1980; Clancy 2009; ISSG 2010). The imbalance of soil nutrients due to gorse invasion may cause long-term effects to natural nutrient levels in the soil profile of invaded sites (Marchante et al. 2009; Lankau et al. 2014; Broadfield and McHenry 2019).

### Agriculture and economy

Common gorse poses a major threat to agriculture and economy in many countries such as New Zealand, Australia and USA (Blaschke et al. 1981; Bascand and Jowett 1982; Hill and Sandrey 1986; Gouldthorpe 2009; ISSG 2010; ODA 2014; Atlan et al. 2015a). The plant was intentionally introduced into multiple ranges mainly for agricultural or ornamental purposes (Holm et al. 1997), however, the plants spread rapidly and became invasive due to the lack effective management (Atlan et al. 2015a). Common gorse is the second most serious weed in New Zealand and has reduced nearly 3.56% of the agricultural land area in South Island (Blaschke et al. 1981; Bascand and Jowett 1982). Unpalatable spiny foliage of the plant has significantly reduced the quality of pastures through the avoidance of grazing animals (cows, deer, and sheep) (Tulang 1992; Richardson and Hill 1998﻿). In addition, agricultural pests such as rabbits, feral cats, house mice, and foxes are, however, attracted to gorse vegetation as it provides shelters for these vertebrate pests. Thus, the *U. europaeus* infestation ultimately affects the productivity of agricultural lands (Gouldthorpe 2009; ISSG 2010).

The control of gorse infestation in agricultural lands creates direct threats to the economy of the countries as they are expensive and not always effective (Hill et al. 2008). Nearly 5.3 million US$ were spent by the Noxious Plants Council in New Zealand in 1984 – 85 to control the common gorse infestation in 232 thousand hectares of agricultural area (Sandrey 1985; Hill and Sandrey 1986). In 2000, 7 million dollars were spent by the Australian government to control common gorse in agriculture and forests (Gouldthorpe 2009). Due to the nature of the gorse plants, management is costly and involves multiple control practices to be implemented continuously for several years, which can cause a significant economic impact for a country (Zabkiewicz 1976; Hill and Sandrey 1986; Clements et al. 2001). According to the Agriculture and Resource Management Council of Australia and New Zealand in 2000, the tourism industry in Australian mainland and Tasmania have been significantly impacted as the common gorse infestation severely affects the natural beauty of wilderness and pastoral areas (Gouldthorpe 2009; ISSG 2010). According to ODA 2014 reports, in 2012, a total of 28 thousand acres of land area in Oregon, USA was infested with common gorse causing $ 441,000 worth of economic loss, while estimated future gorse infestation is 16,580 thousand acres with 205,576 thousand dollars of economic loss. Native ecosystems in Colombia (*i.e.* Cundinamarca and Boyacá) have also been affected by common gorse infestation (Lowe et al. 2004). The farmlands and the potable water were affected in the invaded areas of Colombia (Colombian Ministry of Environment and Sustainable Development reports, 2018). It was documented that thousands of millions of Colombian pesos were required to restore these lands (Camelo 2015; Niño et al. 2018). More studies are needed to understand the economic impact of common gorse invasion on many countries, especially in the tropics.

### Fire hazards

The high amount of oil present in gorse foliage and seeds makes this plant highly flammable (Baeza et al. 2002; Madrigal et al. 2012). The gorse fire is hard to control due to the pyrophilic characteristics of the plant such as quick-burning ability and rapid-fire propagation (Marino et al. 2011; Niño et al. 2018). Thus, the gorse fire causes significant damages to human settlements and forest ecosystems. Fire intolerant native plants are highly vulnerable to the high heat intensity of this feral plant. This would also affect the growth and development of other invasive alien species (Marambe and Wijesundara 2021). A total of 1000 ha of forest plantations were destroyed in both New Zealand and British Colombia due to fires caused by common gorse (Zielke et al. 1992). In 1936, common gorse infestation caused a catastrophic wildfire at Bandon, United State of America, and subsequently, in 1980, 1999, 2007, and 2015, gorse wildfires have done notable destruction to Bandon (GAG 2019). Not only the invasive range, but the native range of the gorse is also affected due to the high flammability of this plant (MacCarter and Gaynor 1980; IPMIS 2000; ARMCAN 2003; Marino et al. 2011). For instance, shrub lands in Galicia, Spain where gorse is native, are under threat due to the intensified gorse wildfires (Marino et al. 2011). The frequent gorse fires due to widespread gorse vegetation in Donegal County, Ireland affect the fauna and flora as well as human activities (DCC 2014).

## Potential benefits of gorse to invaded ecosystems and local species (Fig. 3)

**Fig. 3:**
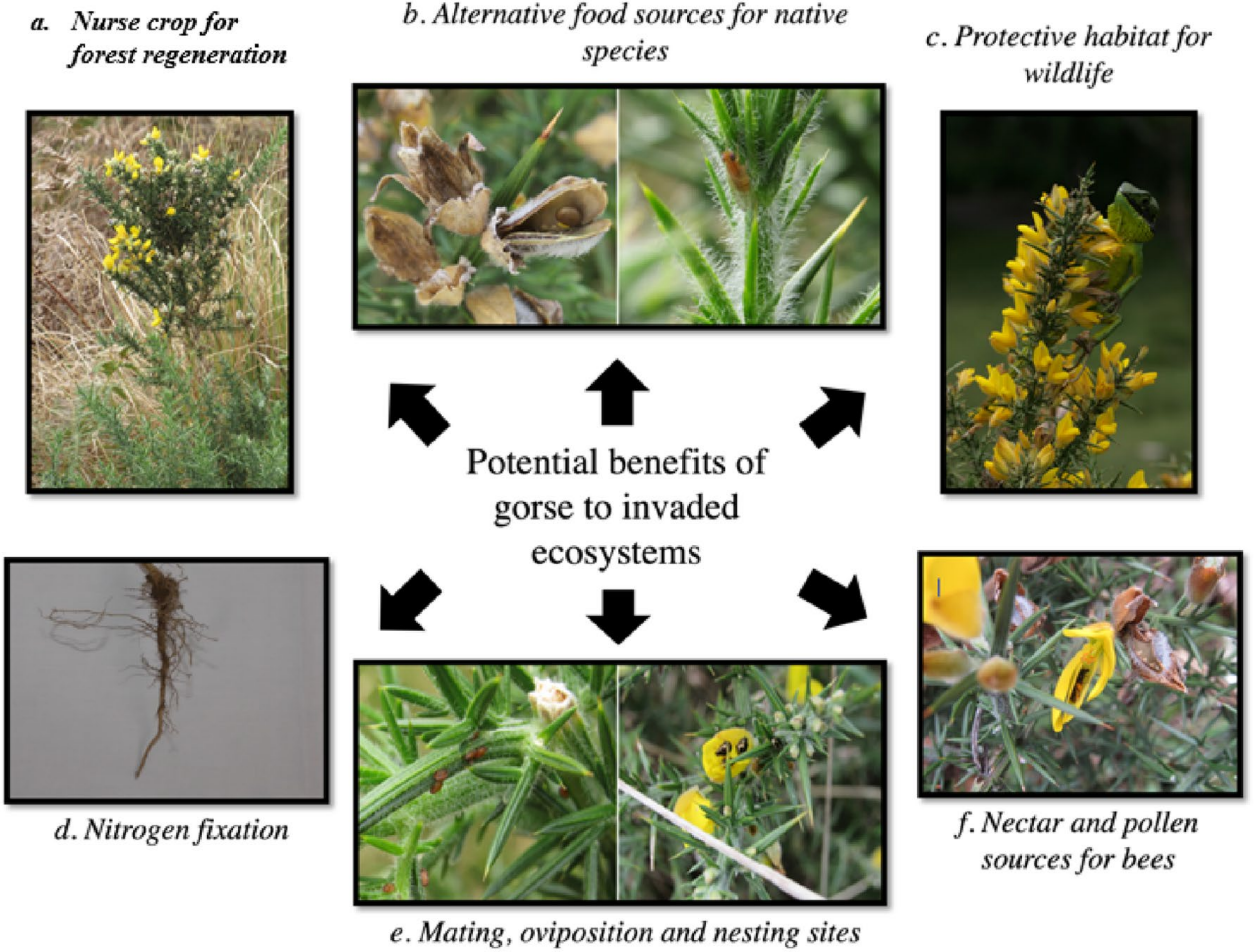
Potential benefits of gorse to invaded ecosystems (PC: a., b., e., and f. Hansani S. S. Daluwatta Galappaththi; c. Dr. Ruchira Somaweera; d. Paul Barret)

### Nitrogen fixation

Nitrogen (N) is the most limiting nutrient for plant growth (Franche et al. 2009). Biological nitrogen fixation plays a key role in N cycling in natural ecosystems (Jensen and Hauggaard-Nielsen 2003). Leguminous and actinorhizal plants are capable of forming nodules inhabited by symbiotic N-fixing bacteria (Franche et al. 2009). Their ability to fix N makes them often the first colonizing species in disturbed soil (McQueen et al. 2006; Goldstein et al. 2010). Among Leguminous plants, *U. europaeus* has been identified to produce a voluminous amount of fixed N through its’ ability of rapid symbiotic N-fixation in nodules (Magesan et al. 2012). Egunjob (1969) found that gorse has an annual rate of 100 – 200 kg ha^–1^ of N accumulation during the dry matter accumulation period. Thus, common gorse contributes to the high input of soil N (Magesan et al. 2012). Therefore, gorse can strengthen the quality of poor soil and soil fertility even in highly disturbed areas (Wardle and Greenfield 1991; Colebatch et al. 2002; Ehrenfeld 2003; Goldstein et al. 2010) although excessive N fixation can negatively impact the ecosystems as reviewed in the previous sections.

### Soil enrichment and as a nurse plant

Common gorse has been considered as a highly successful plant in disturbed soil which would improve the ecological health of invaded sites (Clements et al. 2001). For instance, the plant is used to stabilize sandy soil, roadside banks, maritime areas, and mine waste sites where the soil is less productive. That would increase the soil quality enabling plant propagation and growth (Huxley and Griffiths 1992). This plant can tolerate a wide range of climatic and soil types even disturbed and unproductive geographical areas (Matthews 1982; McAlpine et al. 2009). Gorse produces a higher litter amount that regulates the soil organic matter. This high litterfall increases the available nutrients for plant growth and influences the carbon cycle (Ganjegunte et al. 2005). For instance, 7 – 8 years old gorse plants produce approximately 9000 kg ha^−1^ of annual litterfall at Taita-Kenya (Egunjobi 1969). Lee et al. (1986) found that gorse densities of 60000 stem ha^–1^ produce a litter depth of 55 mm on mature sites in Dunedin, New Zealand.

With aid of high N fixation, the capability of developing, stabilizing, and enriching the poor soil, and early colonization, *U. europaeus* can support forest regeneration, acting as a nurse plant for the restoration of disturbed areas. Especially, the gorse vegetation provides shelter for native seedlings and allows more light penetration to the ground level when they mature. These qualities allow the gorse plant to act as a pioneer transient successional species which ultimately replaced by the native plant species (Druce 1957; Healy 1961; Hackwell 1980; Lee et al. 1986; Wilson 1990a, 1994b; Clements et al. 2001; Harris et al. 2004; Sullivan et al. 2007; CRFRP 2013). For instance, many studies have shown that gorse is a pioneer successional species in disturbed geographical areas by fire, mining, or logging in Australia, China, and New Zealand (Egunjobi 1969; Zabkiewicz 1976; Roberts et al. 1981; Hill et al. 2001; Johnson 2001).

### As a habitat for wildlife

The faunal diversity of a particular habitat would also depend on the soil type, topography, levels of grazing, and drainage of the associated habitat (KWT 2021). Wildlife tends to select a suitable habitat considering food availability, predator pressure, and other factors that affect the survival of their subsequent generations (Somaweera et al. 2012). The presence of few common gorse plants in scrub successional habitats aids in the existence and maintenance of wildlife (KWT 2021). Dense gorse vegetation provides a safe living habitat for wild fauna including birds, reptiles, and invertebrates (Tubbs 1974; Gouldthorpe 2009). A substantial number of studies have been done to discuss the habitat preference and influence of gorse vegetation on different faunal groups and this information is summarized below. However, the available literature is not sufficient to understand the broader impact of gorse vegetation as shelters for wildlife in all regions of the world.

#### Vertebrates

a. Mammals

Common gorse provides food for a wide range of mammals, especially in introduced geographical areas. Bao et al. (1998) explained that *Ulex* was used as a protein source of animal foods. This plant has good digestibility with a useful amount of protein and significant sodium content (Jobson and Thomas 1964; Atlan et al. 2015a). For instance, young shoots of gorse provide an alternative food source for Sambar Deer (*Cervus unicolour*) in Horton Plains National Park, Sri Lanka, which is a vulnerable species native to South and Southeast Asia (Sankar and Acharyal 2004; Somaweera et al. 2012; Timmins et al. 2015). Other livestock such as ponies, horses, cattle, sheep, and goats also consume gorse fodder  in its introduced ranges including Chile, Brazil, Australia, and New Zealand (Thomson 1922; Jobson and Thomas 1964; Tubbs 1974; Radcliffe 1985; Sandrey 1987; Howe et al. 1988; Lambert et al. 1989; Popay and Field 1996; ﻿Norambuena et al. 2000; Parsons and Cuthbertson 2001; Cordero et al. 2016; Broadfield and McHenry 2019﻿). The gorse fodder would strengthen the conditions of horses and the quantity and quality of cow and sheep milk (Atlan et al. 2015b). It is reported that mammals selectively feed on different life stages of the gorse plant. For example, cows preferentially feed on gorse seedlings while sheep and horses have been reported to feed on mature plants (Cordero et al. 2016). Cowan (1990) has carried out a study in New Zealand to find the diet of brushtail possums (*Tnchosurus vulpecula*) that is an introduced species to New Zealand, native to Australia. The study has shown that these marsupials feed on the seeds and the flowers of *U. europaeus*. A subspecies of them is *Trichosurus vulpecula vulpecula* which is considered as a threatened species of the Northern Territory of Australia (Pavey and Ward 2012). Since common gorse is also present in Australia as an invasive weed, this subspecies may also feed on the plant. Thus, it is worthwhile to investigate more about their diet and habitat related to common gorse for the conservation of *T. vulpecula vulpecula*. Further, in case of the plant’s native range, wild herbivores, such as red deer (*Cervus elaphus* L.), roe deer (*Capreolus capreolus* L.), and rabbit (*Oryctolagus cuniculus* L.) (González-Hernandez and Silva-Pando 1996, 1999; Alves et al. 2006), and domestic ungulates, such as goats, sheep, cows, and horses are known to feed on the common gorse plant (Putman et al. 1987; Howe et al. 1988; Clements et al. 2001; Atlan et al. 2015a).

b. Birds

Quails and Chickens are known to associate with common gorse as they prefer to consume gorse seeds (Chater 1931; Clements et al. 2001). Amaya-Villarreal et al. (2010) reported that *Diglossa humeralis* (Black Flower Piercer) and *Basileuterus nigrocristatus* (Black-crested l), which are South American species, mostly prefer and are highly abundant in forest edges, invaded by *Ulex*. According to Carlos and Gibson (2010), a high bird abundance and richness have been recorded in gorse invaded areas in Victoria State, Australia. Despite its invasive nature, the common gorse has created ideal habitats for these avian species. In Tasmania, gorse vegetation has maintained a high forest bird biodiversity by preventing the invasion of the noisy miner (*Manorina melanocephala*) and providing secure ground habitat for nationally vulnerable eastern barred bandicoot (*Perameles gunnii*) (MacDonald 2001; Galea 2003). RSPB (2021) reported that compact common gorse vegetation facilitates ideal nesting sites for birds such as Dartford warbler (*Sylvia undata*), stonechat (*Saxicola rubicola*). Linnet (*Linaria cannabina*), and yellowhammer (*Emberiza citronella*). These habitats provide protective environments during extreme weather conditions (RSPB 2021). Gorse flowers supply year-round nectar and pollen for invertebrates including bees and butterflies (STRI 2021; Woodland Trust 2021).

c. Reptiles

Grown common gorse vegetation provides protective shelters for ectothermic reptiles as such habitats minimize extreme evaporation. Therefore, reptiles such as sand lizards, grass snakes, adders, and smooth snakes are favoring the dense common gorse vegetation (Edgar et al. 2010). The voids among the common gorse roots are known to be used by many reptiles for hibernation while the thorny, prickly edges provide protective basking sites for reptiles (Edgar et al. 2010). For instance, the Black-cheeked Lizard (*Calotes nigrilabris*) is an endemic, nationally threatened and vulnerable lizard in Sri Lanka. This lizard has been found to inhabit common gorse where they have favorable microhabitat conditions (Jayasekara et al. 2019). This lizard experiences less predator pressure under *U. europaeus* bushes as they create dense spiny, vegetation. Further, the body color of the lizard is blended with the gorse plant which protects them from predators (Jayasekara et al. 2019). It has also been shown that *C. nigrilabris* feeds on the honey bees, butterflies, and other insects that visit the gorse plant (Somaweera et al. 2012). The findings of the above studies provide promising information about the use of common gorse habitats by a wide range of reptiles. 

#### Invertebrates-arthropods

A wide range of arthropods, especially, insect assemblages have been identified as associated with *U. europaeus*. Most of the literature extractions provide information of arthropod interactions with the plant in its’ native range (Hill 1982; Stone 1986; Hill et al. 2000; Hill et al. 2001; Hornoy et al. 2013a). The whole plant (roots, leaves, spines, stems, shoots, flowers, and seeds) contains edible parts for both adults and larval stages of arthropods. Dead plant material also provides a great source for detritus utilizing arthropods (Harris et al. 2004). Some of them have been recorded to feed on common gorse exclusively, while generalist feeders may prefer to feed on the plant only when other food is becoming scarce (Hill 1982; Stone 1986; Harman et al. 1996; Hill et al. 2000; Clements et al. 2001; Sixtus 2004; Davies et al. 2005; Hayes 2007; Hornoy et al. 2013a).

This plant provides a range of oviposition and development sites for all insect life stages. Seed pods and plant surfaces especially, leaves and spine axils, mature spines, shoots, slits within young stem are suitable sites for egg deposition and development (Davies 1928; Cowley 1983; ﻿ Markin and Yoshioka 1996; Norambuena et al. 2004; Davies et al. 2005; Andreas et al. 2017). Some arthropods like moth larvae and mites create their webs among branches of the common gorse plant. Especially, terminal branches of the plant are used as a substrate for this fauna for web-spinning (Hill and O'Donnell 1991; Sixtus 2004; Marriott et al. 2013). Some weevils take  advantage of the seed hurling and bursting mechanisms of common gorse. This aids in them to disperse away from the host plant (Davies 1928). It is expected that given the variety of resources offered by gorse, local arthropods in their invasive ranges would make use of these abundant and readily available resources.

Harris et al. (2004) described the arthropod species associated with *U. europaeus*-invaded shrublands in New Zealand. The gorse habitat was species-rich compared with native vegetation (kānuka) for various insect groups including tachinid flies (parasitoids), fungus gnats (detritivores), and beetles (herbivores). Interestingly, some native New Zealand herbivores were only found in the gorse study area, for instance, the lepidopterans *Pyroderces anarithma* Meyrick (Cosmopterigidae), *Eutorna phaulocosma* Meyrick (Depressariidae), *Musotima nitidalis* Walker (Crambidae), *Sestra humeraria* Walker (Geometridae), and the coleopteran *Sharpius brouni* Sharp (Anthribidae). This suggests that some native species may be undergoing host-plant shifts and that some groups benefit particularly from the resources offered by this invasive plant.

Nectar foraging insects, especially honey bees (*Apis mellifera*) and bumblebees (*Bombus terrestris*), have been identified as major pollinators of the common gorse in Western France where the plant is native (Bowman et al. 2008). The plant acts as a rewarding plant for bees as it provides both nectar and pollen (Sixtus 2004; Bowman et al. 2008). Plant nectar supplies majorly carbohydrates for the hives whereas pollen provides protein and vitamin requirements for the hives (Koning 1994). Gorse pollen is majorly used by bees to feed their larval stages (Bowman et al. 2008). However, more studies need to be done in order to identify the arthropods associated with invaded tropical countries and other temperate regions.

#### Fungi and microorganisms

Johnston et al. (1995) have described more than 20 fungal species that use gorse as a host plant for their life cycle. *Uromyces pisi* is one of them and subspecies have been identified as relatively specific to the gorse plant in the native range of the plant (Hill et al. 2000, 2008; Andreas et al. 2017). Further, it is a well-known fact that gorse harbors symbiotic N-fixing bacteria (Franche et al. 2009). However, very little is known about gorse-microorganisms/fungal interactions in its invaded ranges. Further investigations are required to address this knowledge gap.

### Human uses

In the past, common gorse flowers were used to prepare colorings, while seeds were used to produce pesticides to control fleas (Grieve 1984). These fire-prone plants were used for kindling and heating the ovens. The ashes of the burnt wood are fertilizer for plant growth. They are rich in Potassium and mixed with vegetable oils or clay to produce soaps. Especially in Caldey Island, Wales, UK, this plant is used to produce, perfumes, soaps, and bath oils (Johnson and Sowrby 1899; Bean 1981; Grieve 1984; Freethy 1985; Miller and Murthy 2009). Common gorse makes spiny thickets that can face external forces very well. Thus the plant has been used as hedges for shelter and as a barrier to the wind forces especially in maritime ranges (Rosewarne Experimental Horticultural Station 1984; Hill and Sandrey 1986; Huxley and Griffiths 1992; Magesan et al. 2012). Some parts of the common gorse are apt for human consumption. The flower buds are used to make pickles with vinegar that is preferably used like capers in salads (Facciola 1990), while shoot tips are consumed as tea (Kunkel 1984; Facciola 1990). Gorse pollen plays a vital role in the bee-keeping industry. Brood rearing of honeybee colonies has been proven to be entirely dependent on the gorse pollen as the plant produces high-quality pollen throughout the year (Sandrey 1985). Hill and Sandrey (1986) have described that beekeepers may suffer economically if the *U. europaeus* plant is highly controlled in areas where it is integrated with apicultural practices.

Further, common gorse has drawn interest due to its medicinal, immunological, and biochemical values. Flowers have been used to treat jaundice and scarlet fever in children. Seeds are used against diarrhea and gall stones (Grieve 1984). The plant is also used in Bach Flower Remedies (Chancellor 1985). The Lectin extraction from the *U. europaeus* seeds known as *Ulex europaeus* I agglutinin (UEA I) has a high demand in immune biology. It is used to determine the A and AB blood groups, diagnose the secretor status, and as a marker for human endothelium vascular tissue lesions (Boyd and Shapleigh 1954a, 1954b; Holthöfer et al. 1982; Jackson et al. 1990; Uchida et al. 1997; Rodd and Boissonade 2005; Clini Sciences 2021).

The volatile compounds of the *Ulex* plant give a high fuel load especially through their branches and litter layer (Anderson and Anderson 2009). A total of 46 – 52 t ha^−1^ of fuel load has been calculated within common gorse shrublands in Spain (Vega et al. 2005). In New Zealand, a total of 26 – 74 t ha^−1^ of fuel load has been reported from a range of gorse sampling sites (Anderson and Anderson 2009). Núñez-Moreno et al. (2020) has evaluated the potential use of the common gorse plant as a biofuel in Colombia. According to the findings of their study, the generated solid biofuel of common gorse has 75% carbon heat value, 83.3% of highly volatile material content, and 1.41% and 0.15% of ash and Sulphur residues, respectively. Therefore there is a possibility of biofuel production using common gorse that would be an alternative eco-friendly renewable energy source particularly in countries that have fuel shortages.

Ligero et al. (2011) reported that the common gorse plant contains 12% of Xylose, suggesting its use as a promising biomass source to extract Xylan-associated compounds. These compounds are widely used in food, plastic, papermaking, and textile printing industries as a thickener, additive, emulsifier, protein foam stabilizer, and a food preservative (Ebringerová et al. 1994, 1995; Hromadkova et al. 1999; Ünlü et al. 2009; Li et al. 2011). The extractions of aerial parts (flowers, leaves) and root barks of *U. europaeus* consist of phenolic compounds such as flavone, isoflavones, and flavanones that have high pharmacological relevance (Spínola et al. 2016). Minor amounts of other phenolic acids such as caffeic, coumaric, ferulic, and saponins have also been detected from this plant (Russell et al. 1990; Máximo et al. 2002; Spínola et al. 2016). These phenolic compounds have insecticidal and antioxidant properties (Máximo et al. 2002; Lopez-Hortas et al. 2016). Moreover, the phenolic compounds present in the common gorse suggest the bio-herbicidal properties of this plant. In in vitro bioassays, the extraction from the flowering foliage of common gorse inhibited the germination and early growth of agricultural weeds *i.e. Amaranthus retroflexus* and *Digitaria sanguinalis* (Pardo-Muras et al. 2020).

In addition, Tighe-Neira et al. (2016) described the potential use of aqueous extraction of the common gorse as a fortificant in agronomy. Extractions of *U. europaeus* stimulate the green and dry matter of *Capsicum annuum* L. seedlings. The plant can also be used to obtain biochar through pyrolysis which is useful to purify wastewater. For instance, common gorse biochar has been proven as an effective sorbent for chromium in Bogotá-Colombia River Water (Gomez et al. 2021). Moreover, Celis et al. (2014) and Pesenti et al. (2017) have assessed the capability of production of bio-fiber/biopolymer using *U. europaeus* in Chile while Bonilla and Bonilla (2021) have synthesized novel lignin-based biopolymer in Colombia. These studies proved that the biopolymer of common gorse is thermally stable and has a high degree of crystallinity. An interesting study done by Jobson and Thomas in 1964 reported that the common gorse plant contains crude protein (13.6%), fat (1.9%), nitrogen (46.3%), fiber (34.7%), ash (3.5%), and silica (0.3%). Miller and Murthy (2009) have tested and proved the plant’s potential use in the production of oil, ethanols, hexanes, and dyes. Spectrophotometric analysis of the above study showed the high light absorption levels at visible wavelengths depicting the plant’s potential use in the production of dyes.

## Genetic diversity and evolution of life-history traits of common gorse

The environmental and ecological factors in introduced areas are different from the native ranges of any introduced species. The existence and the colonization of exotic species in novel habitats are assured by the phenotypic plasticity and the adaptability of any organism (Davidson et al. 2011; Ebeling et al. 2011; Zhao et al. 2013; Griffith et al. 2014). Many studies have been done to understand the genetic diversity and evolution of life history which influences the persistence of invasive species in introduced geographical ranges (Hornoy et al. 2013b; Udo et al. 2017). According to Hornoy et al. (2013b), the genetic diversity within the population of common gorse is significantly high. This study has emphasized that the introduction of common gorse into new geographical areas resulted in the loss of some rare alleles and the reduction of genetic diversity. For instance, a significant reduction in the genetic diversity was reported when the plant naturally spread out from Spain towards northern Europe (Hornoy et al. 2013b). In addition, Hozawa and Nawata (2020) have reported the genetic diversity of common gorse in Maui, California, Hawaii, and New Zealand. These authors reported that the most similar genetic diversity of *U. europaeus* sampled in these four introduced regions. Studies have reported the genetic variations associated with seedlings and seed mass of common gorse as well (Hornoy et al. 2011; Atlan et al. 2015a; Udo et al. 2017). A study done by Udo et al. (2017) compared the seed germination strategies of common gorse in the native range (France) and an invaded area (Reunion). The results have shown the faster germination of the variety from the invaded area. Atlan et al. (2010) reported the genetic differences between flowering types of common gorse. These studies further reported the evolution of life-history traits that enhance the fitness of these plants. Atlan et al. (2015c) have assessed the phenotypic plasticity of common gorse in reproductive traits response to shading. The results of this study have found that the dense shade decreases flower and pod production of common gorse**.**

## Mapping aspects of common gorse

Due to the high impact of invasive species on the economy, ecology, and the environment of the invaded country (Pimentel et al. 2000; Simberloff 2011; Paz-Kagan et al. 2019), predictions of the negative impact of the invasive species are crucially important in minimizing the ecological as well as economic impacts (Early and Sax 2014; Paini et al. 2016; Bekele et al. 2018). The early detection and prevention of invasive species spreading are vital to ensure biosecurity as well (Zimmermann et al. 2007; Pyšek and Richardson 2010; Paz-Kagan et al. 2019). For early detection and also to propose action plans in mitigating negative impacts, species distribution modelings (SDM) together with satellite images and remotes sensing data have taken an increased interest (Phillips et al. 2006; Elith and Leathwick 2009; Gränzig et al. 2021). Mapping of the distribution of common gorse in diverse geographical areas is, therefore, in high demand (Gomes et al. 2018; Thapa et al. 2018; Gränzig et al. 2021). Many studies have already reported the significant contribution of GIS and other mapping systems in minimizing gorse invasion. For instance, Gränzig et al. (2021) has shown the potential application of Sentinel-2 imagery and the unmanned aerial vehicles (UAV) orthoimages to determine the distribution patterns of common gorse in Chile. The investigation based on SDM has been done by Christina et al. (2020) to predict the climatic niche changes of common gorse in the native range and the introduced areas. The findings forecast the niche expansion of common gorse in 49%, 111%, 202%, and 283% in Australia, North Europe, North-West America, and South America, respectively. Kariyawasam and Ratnayake (2019a) have done a Maxent model-based study for the common gorse distribution in South Australia and Sri Lanka. According to these findings, common gorse is predicted to be distributed widely in the Mount Lofty Ranges and Kangaroo Island areas of South Australia. Further, Rees and Hill (2001) developed a model to determine the biological control of common gorse via seed-feeding. The available literature suggests that the integration of GIS and mathematical modeling in understanding the invasion potential of invasive species is an emerging but important field of science (Kariyawasam et al. 2021).

## Invasive and native common gorse: a comparative analysis

The comparative studies of invasive species in their native/introduced area are important to understand the effect of ecological and socioeconomic impacts as an invasive species to the invaded ecosystems (Vilà et al. 2010). Such studies have been extensively done for many invasive species (Hinz and Schwarzlaender 2004; Flores-Moreno et al. 2013), but only a few studies have been done for common gorse. According to the study conducted by Medina-Villar et al. (2021), the physical defenses of common gorse in its invaded range (Chile) are significantly higher as compared to the native range (Spain). This study suggests biomass and the size of the thorns of *U. europaeus* are higher in the invaded range. Further, the spine density of the seedling stages in the invaded range is comparatively higher than in the native range. The outcomes of these studies are explained by the Enemy Release Hypothesis (ERH) which is the lower pressure of herbivores in the invaded areas drives the low investment in physical defenses while giving priority to growth and reproduction. Similar observations were reported from the studies done by Hornoy et al. (2011, 2012). In accordance with this study, the seedlings of the common gorse plant in the invaded ranges were taller compared to the native range. However, the insect infestation rates and defensive alkaloids concentrations were observed to be similar in both invaded and native regions. La Pierre et al. (2017), has compared the rhizobia association of three invasive legumes (*Genista monspessulana*, *Spartium junceum*, and *Ulex europaeus*) and six native legumes (*Acmispon glaber*, *A. heermannii*, *A. micranthus*, *A. strigosus*, *Lupinus arboreus*, and *L. bicolor*) in the San Francisco, California, USA. The outcome of this study reported that common gorse does not have an association with the mutualists of local native legumes of San Francisco region, although there is a possibility for such formations.

Morais et al. (2012) conducted a study to evaluate the salinity tolerance capability of *U. europaeus* (native to Portugal) and *Acacia longifolia* (invasive to Portugal). This study reported that salinity tolerance ability of *U. europaeus* is relatively less in its native range than when it is co-occurring with the invasive *Acacia longifolia*. Moreover, a comparative study done to compare the plant vegetative size and soil seed bank of *U. europaeus* in its native range and the invaded ranges showed that relatively larger seed banks in invaded ranges than on the native range (Bakker et al. 2019). Larger maternal plant size, lower activity of seed predators, and higher soil fertility in the invaded areas were suggested as the potential reasons for these variations. According to Atlan et al. (2015a), the common gorse plant shows similar self-fertility levels in both native and invaded ranges whereas seed mass and the seed germination rate is relatively high in invaded areas compared to the native regions.

## Discussion and research needs

Table 1 summarizes the research work that has been done to understand the ecology and biology of common gorse as well as the assessment of invasion of the plant to diverse ecosystems and the control measures that have been taken so far

**Table 1:** Summary of the literature review of biology, ecology, and invasiveness of common gorse (*U. europaeus*)

Reference No	Country/Region	Research Topic	Research Area	References
1	Global	A global view of the future for biological control of gorse, *Ulex europaeus* L.	Biological control of gorse	Hill et al. (2008)
2	Global	The invasive niche, a multidisciplinary concept illustrated by gorse (*Ulex europaeus*)	Identifying the status of gorse in different countries	Atlan and Udo (2019)
3	Review	A world of gorse: persistence of *Ulex europaeus* in managed landscapes	Gorse ecology and management	Broadfield and McHenry (2019)
4	Global	Climatic niche shift of an invasive shrub (*Ulex europaeus*): a global scale comparison in native and introduced regions	Niche shift and distribution maps	Christina et al. (2020)
5	Review	Biology, distribution, and control of the invasive species *Ulex europaeus* (Gorse): A global synthesis of current and future management challenges and research gaps	Gorse biology, distribution, and management	Roberts and Florentine (2021)
6	New Zealand	The bionomics of *Apion ulicis* Först (gorse weevil), with special reference to its role in the control of *Ulex europaeus* in New Zealand 1	Biological control of gorse	Davies (1928)
7	New Zealand	A contribution to the study of the natural control of gorse	Natural control of gorse	Chater (1931)
8	New Zealand	Dry matter and nitrogen accumulation in secondary successions involving gorse (*Ulex europaeus* L.) and associated shrubs and trees	Gorse as a pioneer successional species	Egunjobi (1969)
9	New Zealand	The ecology of gorse and its relevance to New Zealand forestry	Gorse ecology	Zabkiewicz (1976)
10	New Zealand	Gorse: a subject for biological control in New Zealand	Biological control of gorse	MacCarter and Gaynor (1980)
11	New Zealand	Life cycle of *Apion ulicis* (Coleoptora: Apionidae) and gorse seed attack around Auckland, New Zealand	Biological control of gorse	Cowley (1983)
12	New Zealand	Gorse control in New Zealand forestry-the biology and the benefits	Gorse biology, benefits and control	Zabkiewica (1984)
13	New Zealand	Grazing management of goats and sheep for gorse control	Biological control of gorse	Radcliffe 1985
14	New Zealand	Biological control of gorse, an ex-ante evaluation	Biological control of gorse	Sandrey (1986)
15	New Zealand	Gorse and goats: considerations for biological control of gorse	Biological control of gorse	Sandrey (1987)
16	New Zealand	The costs and benefits of gorse	Costs and benefits of gorse	Hill and Sandrey (1986)
17	New Zealand	Succession and dynamics of gorse (*Ulex europaeus* L.) communities in the dunedin ecological district South Island, New Zealand	Gorse as a pioneer successional plant	Lee et al. (1986)
18	New Zealand	Voluntary intake and digestion of gorse (*Ulex europaeus*) by goats and sheep	Gorse biochemistry	Howe et al. (1988)
19	New Zealand	Control of gorse in hill country: an economic assessment of chemical and biological methods	Gorse control	Krause et al. 1988
20	New Zealand	Forage shrubs in North Island hill country 1. Forage production	Impacts on the ecosystem	Lambert et al. (1989)
21	New Zealand	Isoflavones from root bark of gorse	Gorse biochemistry and human uses	Russell et al. (1990)
22	New Zealand	Gorse on Hinewai Reserve	Impacts on the ecosystem	Wilson (1990a)
23	New Zealand	Fungi associated with gorse and broom in New Zealand	Gorse-fungal associations	Johnston et al. (1995)
24	New Zealand	Arthropod introductions for biological control of weeds in New Zealand	Biological control of gorse	Harman et al. (1996)
25	New Zealand	Mechanisms of *Pinus radiata* growth suppression by some common forest weed species	Impacts of gorse on growth suppression of introduced plants	Richardson et al. (1996)
26	New Zealand	The biological control program against gorse in New Zealand	Biological control of gorse	Hill et al. (2000)
27	New Zealand	Large-scale disturbances, biological control and the dynamics of gorse populations	Biological control/modeling	Rees and Hill (2001)
28	New Zealand	Vegetation recovery after fire on a southern New Zealand peatland	Vegetation recovery after fire	Johnson (2001)
29	New Zealand	Insect assemblages in a native (kanuka–*Kunzea ericoides*) and an invasive (gorse–*Ulex europaeus*) shrubland	Gorse-insect associations	Harris et al. (2004)
30	New Zealand	An investigation of the life history of the gorse pod moth (*Cydia succedana*) and its effectiveness at reducing gorse (*Ulex europaeus*) seed production	Biological control of gorse	Sixtus (2004)
31	New Zealand	Secondary forest succession differs through naturalised gorse and native kānuka near Wellington and Nelson	As a pioneer successional plant	Sullivan et al. (2007)
32	New Zealand	Flexible boundaries in biosecurity: accommodating gorse in Aotearoa New Zealand	Biosecurity study	Barker 2008
33	New Zealand	Invasive legumes fix N_2_ at high rates in riparian areas of an N‐saturated, agricultural catchment	Nitrogen fixation	Drake (2011)
34	New Zealand	Nitrogen cycling in gorse-dominated ecosystems in New Zealand	Nitrogen fixation	Magesan et al. (2012)
35	New Zealand	Catchment-scale contribution of invasive nitrogen fixing shrubs to nitrate leaching: a scoping study	Nitrogen fixation	Mason et al. (2016)
36	Canada	Broom and gorse: a forestry perspective problem analysis	Impacts on the forests	Zielke et al. 1992
37	Canada	The biology of Canadian weeds 112 *Ulex europaeus* L	Gorse biology and impacts on the ecosystem	Clements et al. (2001)
38	Canada	Predicting the elevated dead fine fuel moisture content in gorse (*Ulex europaeus* L.) shrub fuels	Predicting the fuel moisture content of gorse	Anderson and Anderson (2009)
39	Sri Lanka	Does the invasive shrub *Ulex europaeus* benefit an endemic Sri Lankan lizard	Gorse as a habitat for Sri Lankan lizard	Somaweera et al. (2012)
40	Sri Lanka	Microhabitat Utilisation of Endemic Lizard *Calotes nigrilabris* in the Grasslands of Horton Plains National Park, Sri Lanka	Gorse as a habitat for Sri Lankan lizard	Jayasekara et al. (2019)
41	South Australia, Sri Lanka	Invasive ranges of *Ulex europaeus* (Fabaceae) in South Australia and Sri Lanka using species distribution modeling	Distribution and Mapping of common gorse	Kariyawasam and Ratnayake (2019a)
42	South Australia, Sri Lanka	Reproductive biology of gorse, *Ulex europaeus* (Fabaceae) in the mount lofty ranges of South Australia and Sri Lanka	Gorse biology	Kariyawasam and Ratnayake (2019b)
43	Australia	Binding of human endothelium to Ulex europaeus I-coated Dynabeads: application to the isolation of microvascular endothelium	Immunological uses	Jackson et al. 1990
44	Australia	The biology of Australian weeds. 34. *Ulex europaeus* L.	Gorse biology	Richardson and Hill (1998)
45	Australia	The habitat value of gorse *Ulex europaeus* L. and hawthorn *Crataegus monogyna* jacq. for birds in Quarry Hills bushland park, Victoria	Gorse-bird associations	Carlos and Gibson (2010)
46	Australia	Effects of *Tetranychus lintearius* (Acari: Tetranychidae) on the structure and water potential in the foliage of the invasive *Ulex europaeus* (Fabaceae) in Australia	Gorse- *Tetranychus lintearius* associations	Marriott et al. (2013)
47	Tasmania	Response of small mammals to site characteristics in the Northern Midlands of Tasmania	Gorse-small mammal associations	Galea (2003)
48	Tasmania	The impact of gorse thrips, ryegrass competition, and simulated grazing on gorse seedling performance in a controlled environment	Biological control of gorse	Davies et al. (2005)
49	Tasmania	Gorse-national best practice manual	Gorse biology and control	Gouldthorpe 2009
50	South Africa	Scotch broom (*Cytisus scoparius* (L.) link) and gorse (*Ulex europaeus* L.) in South Africa: an assessment of invasiveness, management options and feasibility for countrywide eradication	Assessment of invasiveness and management of gorse	Mbatha (2016)
51	Hawaii	Introduction and establishment of the biological control agent *Apion ulicis* (Forster) (Coleoptera: Apionidae) for control of the weed gorse (*Ulex europaeus* L.) in Hawaii	Biological control of gorse	Markin and Yoshioka (1996)
52	Hawaii	The major features of an infestation by the invasive weed legume gorse (*Ulex europaeus*) on volcanic soils in Hawaii	Ecological features of gorse	Leary et al. (2006)
53	USA	Diagnosis of subgroups of blood groups A and AB by use of plant agglutinins (lectins)	Gorse biochemistry and human uses	Boyd and Shapleigh (1954a)
54	USA	Separation of individuals of any blood group into secretors and non-secretors by use of a plant agglutinin (lectin)	Gorse biochemistry and human uses	Boyd and Shapleigh (1954b)
55	USA	Recovering valuable products from Gorse (*Ulex europaeus*)	Human uses—oil and volatile extractions	Miller and Murthy (2009)
56	USA	Biology and biological control of common gorse and scotch broom	Gorse biology and controlling	Andreas et al. (2017)
57	USA	Invasive legumes can associate with many mutualists of native legumes, but usually do not	Gorse-mutualistic associations	La Pierre et al. (2017)
58	Chile	The biocontrol of gorse, *Ulex europaeus*, in Chile: a progress report	Biological control of gorse	Norambuena et al. (2000)
59	Chile	Release strategies for the moth *Agonopterix ulicetella* in the biological control of *Ulex europaeus* in Chile	Biological control of gorse	Norambuena et al. 2004
60	Chile	Characterizing cellulosic fibers from *Ulex europaeus*	Fiber production from gorse	Celis et al. (2014)
61	Chile	The invasive species *Ulex europaeus* (Fabaceae) shows high dynamism in a fragmented landscape of south-central Chile	Landscape characteristics with gorse distribution	Altamirano et al. (2016)
62	Chile	Effects of extracts of *Ulex europaeus* L. on the biomass production in chilipepper (*Capsicum annuum* L.) seedlings, under laboratory conditions	As a growth promoter of biomass production of the plants	Tighe-Neira et al. (2016)
63	Chile	Exploring *Ulex europaeus* to produce nontoxic binderless fibreboard	Fibreboard production	Pesenti et al. (2017)
64	Chile	Mapping the fractional coverage of the invasive shrub *Ulex europaeus* with multi-temporal Sentinel-2 imagery utilizing UAV orthoimages and a new spatial optimization approach	Distribution and Mapping of common gorse	Gränzig et al. (2021)
65	Chile/Spain	The green thorns of *Ulex europaeus* play both defensive and photosynthetic roles: consequences for predictions of the Enemy Release Hypothesis	Ecology and ERH hypothesis	Medina-Villar et al. (2021)
66	Southern Brazil	Invasive gorse (*Ulex europaeus*, Fabaceae) changes plant community structure in subtropical forest–grassland mosaics of southern Brazil	Impacts of gorse on the plant communities	Cordero et al. (2016)
67	Southern Brazil	Analyzing the landscape characteristics promoting the establishment and spread of gorse (*Ulex europaeus*) along roadsides	Landscape characteristics and gorse distribution	León Cordero et al. (2016)
68	Colombia	Effects of gorse (*Ulex europaeus*) on the birds of a high Andean forest edge	Gorse-bird associations	Amaya-Villarreal and Renjifo (2010)
69	Colombia	Evaluation of the current successional stage of restored areas previously invaded by *Ulex europaeus* L.	As a pioneer successional species	Camelo (2015)
70	Colombia	Evaluation of the energy potential of the gorse (*Ulex europaeus*) in the generation of electrical energy by gasification	Potential energy generation	Niño et al. (2018)
71	Colombia	Analysis of the feasibility of generating solid biofuel from *Ulex europaeus* plants	Feasibility of biofuel production	Núñez-Moreno et al. (2020)
72	Colombia	Synthesis and characterization of a novel Lignin-based biopolymer from *Ulex europaeus*: A Preliminary Study	Biopolymer production	Bonilla and Bonilla (2021)
73	Colombia	Use of the Biochar obtained by slow pyrolysis from *Ulex europaeus* in the removal of total Chromium from the Bogotá-Colombia River Water	Biochar production	Gomez et al. (2021)
74	UK	Mechanism of acidification of soil by *Calluna* and *Ulex* and the significance for conservation	Soil acidification	Grubb and Suter (1971)
75	England	The composition of gorse (*Ulex europaeus*)	Gorse composition and biochemistry	Jobson and Thomas (1964)
76	London	The phytophagus fauna of gorse (*Ulex europaeus* L.) and host plant quality	Gorse-phtophagous fauna associations	Hill (1982)
77	Spain	Soil water balance as affected by throughfall in gorse (*Ulex europaeus*, L.) shrubland after burning	Impacts on the soil water quality	Soto and Diaz-Fierros (1997)
78	Spain	*Ulex europaeus* as a protein source for the agrifood industry in Galicia, Spain	As a protein source for agrifoods	Bao et al. (1998)
79	Spain	Throughfall, runoff and soil erosion after prescribed burning in gorse shrubland in Galicia (NW Spain)	Impacts on the soil profile	Vega et al. (2005)
80	Spain	Evaluation of the flammability of gorse (*Ulex europaeus* L.) managed by prescribed burning	Flammability evaluation	Madrigal et al. (2012)
81	Spain	Gorse (*Ulex europaeus*) as a possible source of xylans by hydrothermal treatment	As a possible source of xylans	Ligero et al. (2011)
82	Spain	Fire hazard after prescribed burning in a gorse shrubland: implications for fuel management	Fire hazards	Marino et al. (2011)
83	Spain	Flowers of *Ulex europaeus* L. Comparing two extraction techniques (MHG and distillation)	Extraction of gorse flower content	Lopez-Hortas et al. (2016)
84	Spain	Water-soluble phenolic acids and flavonoids involved in the bioherbicidal potential of *Ulex europaeus* and *Cytisus scoparius*	As a bio herbicide	Pardo-Muras et al. (2020)
85	Brittany, France, Scotland, UK, Reunion Island, New Zealand	Invasive plants and enemy release: evolution of trait means and trait correlations in *Ulex europaeus*	Evolutionary/ Comparative study	Hornoy et al. (2011)
86	Brittany, Scotland Reunion Island, New Zealand	Alkaloid concentration of the invasive plant species *Ulex europaeus* in relation to geographic origin and herbivory	Study of Alkaloid concentration	Hornoy et al. (2012)
87	Brittany, Scotland, Reunion and New Zealand	Oviposition decision of the weevil *Exapion ulicis* on *Ulex europaeus* depends on external and internal pod cues	Gorse-weevil associations	Hornoy et al. (2013a)
88	Spain, Brittany, Scotland, Chile, New Zealand, Reunion Island, USA	Two colonisation stages generate two different patterns of genetic diversity within native and invasive ranges of *Ulex europaeus*	Genetic/Comparative study	Hornoy et al. (2013b)
89	France, New Zealand, Reunion Island	Explaining the larger seed bank of an invasive shrub in non-native versus native environments by differences in seed predation and plant size	Comparative study	Bakker et al. (2019)
90	Brittany, Scotland, New Zealand, Reunion Island	Self incompatibility in *Ulex europaeus*: variations in native and invaded regions	Comparative study	Atlan et al. (2015a)
91	Brittany, Reunion Island	Evolution of the uses of gorse in native and invaded regions: what are the impacts on its dynamics and management?	Uses of gorse in native and invasive range	Atlan et al. (2015b)
92	France	How is the invasive gorse *Ulex europaeus* pollinated during winter? A lesson from its native range	Gorse pollination	Bowman et al. (2008)
93	France	Genetic variation in flowering phenology and avoidance of seed predation in native populations of *Ulex europaeus*	Genetic study	Atlan et al. (2010)
94	France	Phenotypic plasticity in reproductive traits of the perennial shrub *Ulex europaeus* in response to shading: a multi-year monitoring of cultivated clones	Evolutionary study	Atlan et al. (2015c)
95	France, Reunion	Evolution of germination strategy in the invasive species *Ulex europaeus*	Evolutionary study	Udo et al. (2017)
96	Sweden	*Ulex europaeus* I lectin as a marker for vascular endothelium in human tissues	Immunological uses	Holthöfer et al. (1982)
97	Portugal	Flavonoids from *Ulex airensis* and *Ulex europaeus* ssp. *europaeus*	Gorse biochemistry and Flavonoids extraction	Máximo et al. (2002)
98	Portugal	Salt tolerance traits increase the invasive success of *Acacia longifolia* in Portuguese coastal dunes	Salt tolerance ability of *Ulex europaeus* and *Acacia longifolia*/Comparative study	Morais et al. (2012)
99	Portugal	*Ulex europaeus*: from noxious weed to source of valuable isoflavones and flavanones	As a source of valuable isoflavones and flavanones	Spínola et al. (2016)

In this review, we summarize the outcomes of the studies on *U. europaeus* (Table 1). Many studies have been done to determine the biology and invasiveness of gorse plants (~ 27%). A significant number of studies have discussed the different control strategies against the plant as a major invasive weed around the globe (Table 1: 1, 7, 49 and 59). A considerable amount of studies have been done to understand the impacts of common gorse on the ecosystems (~ 31%) and their potential human uses (~ 23%) (Table 1). There is ample evidence from the previous studies to evaluate the negative impact of common gorse on various ecosystems (*i.e.* change of soil profile, effect on natural water bodies and water quality, fire hazards, invasion to agricultural lands, and threats to flora and fauna). Previous studies have also discussed the impact on the economy via mitigations plans in eradicating and controlling *U. europaeus* (Table 1: 35, 36 and 66). However, some studies have clearly discussed the beneficial role of this plant in disturbed ecosystems as a pioneer successional species (plays a significant role in rebuilding the health of disturbed soils), the survival and fitness of native flora and fauna (providing habitats, shelter, food, and protection) (Table 1: 8, 29 and 39). Although currently, *U. europaeus* is considered as a major invasive plant, it was previously introduced into new regions due to its potential value for human uses. The plant has a demand for human food and livestock fodder due to its high amount of nutritional components. Mature plants can sustain against high wind forces as they grow into dense spiny thickets. The *U. europaeus* thickets, therefore, are still widely used for hedge and fencing purposes. Furthermore, the plant is useful for many manufacturing companies due to its medicinal and pharmacological value. The chemical extracts of the plant parts are used in many industries for the production of soaps, fragrances, and oils. Biochar, biopolymer, biofuel production are recent additions to industrial uses (Table 1: 55, 71, 72, 73 and 84).

The success of common gorse in introduced ranges is strongly associated with its remarkable life-history traits including genetic diversity, large seed bank, rapid seed germinations, and fast growth rates even in disturbed soils (Table 1: 89 and 93). Some studies have been done to compare the life-history traits of *U. europaeus* in native and invaded rages (Table 1: 90, 91 and 98), however, more studies are needed for a proper understanding of the adaptive traits of this plant. A few studies have been conducted on the modeling of distribution patterns of this plant (Table 1: 4, 5 and 64). A significant number of studies have been done in New Zealand, followed by Spain, Chile, and Australia to understand the biology, ecology, life-history traits, and control of this plant, however, more studies are needed in tropical ranges where the plant is widely distributed.

This review, based on the available literature, suggests that there are significant knowledge gaps in understanding the positive and negative effects of common gorse on invaded ecosystems. Below, we briefly identify some areas that need to be further explored.

More studies are required to determine the effect of common gorse on the health of the soil and the soil fauna. Studies aiming at the impact of common gorse on the nutrient cycling of the soil and the diversity, distribution, and abundance of soil fauna that are associated with the gorse roots are topics needed to be discussed. Such studies will provide crucial information about the population structure of soil fauna in common gorse invaded areas. Furthermore, the impact of common gorse and its derivatives on the pathogens that are associated with native plants is also an important topic to be investigated. There are also gaps in understanding the interactions between the microorganisms such as fungi and bacteria with the invaded common gorse.

More studies on flowering and nectar production of common gorse plants and the interactions with honeybees and native pollinators are crucial for the bee-keeping industry. Competing traits of this plant and the identification of co-existing native plants are another important aspect that needs more attention. A deep understanding of the interactions of common gorse with the animal communities in the ecosystem needs further attention. Such studies are important in understanding the effect of common gorse on endemic, endangered, and rare organisms in the native range. The biochemistry of the plant as well as the genetic composition which allows this plant to become a successful competitor in diverse ecological conditions are also poorly understood. More studies on the biochemistry of this plant will allow the identification of any toxic effects of the plant derivatives on the native organisms in the introduced range. Investigations on the bioactive compounds of this plant will be useful for drug production, vaccine development, and other pharmaceutical uses. Additionally, the economic importance of the common gorse as fuel, food, or medicine is yet to be addressed critically.

Furthermore, more systematic studies are important for effective control of common gorse in invaded lands. Continuous monitoring plans are required to assess the impact of gorse invasion in invaded ranges. Identification of grazing animals and quantifying the feeding rate of those grazers on different stages of common gorse will be useful in recommending grazing animals as a biological control agent. Integrated approaches which include physical, biological, and chemical approaches should be appropriately designed and planned to manage the adverse effect of common gorse on invading ecosystems, while considering the impacts of control on native species that may benefit from gorse's presence. Few studies have been done to compare the life-history traits of *U. europaeus* in native and invaded ranges and more studies are needed for a proper understanding of the adaptive traits of this plant in different geographic and environmental conditions. The overall impact of invasive species may depend on distribution, local abundance, and per capita effect on the environment (Parker et al. 1999). Several tools such as the Generic Impact Scoring System (GISS) and Environmental Impact Classification of Alien Taxa (EICAT) have been developed to quantify and compare the impact of alien species (Vilà et al. 2019; Lapin et al. 2021). Such methods can be applied to quantify and assess the overall effect of common gorse invasiveness to different regions of the world. Therefore, more studies on distribution patterns along with the climatic and geographical variables as well as landscape patterns are needed, especially on tropical regions.

## Conclusion

Through this paper, we aimed to explore both harmful effects and beneficial roles of common gorse in its invaded ecosystems and potential human uses. The findings suggest that despite its negative impacts, *Ulex europaeus* can also benefit the ecosystems it invades. The plant fixes nitrogen and acts as a nursing plant, allowing degraded native habitats to regenerate. When considering the plant morphology, the whole plant can be described as a source of food, shelter, and source for breeding and development for a wide range of fauna. Moreover, common gorse has a high demand for human uses as well. The plant has drawn the interest of people due to its economic, pharmacological, immunological, medicinal, and edible values. However, studies related to interactions of wildlife with the common gorse in the invaded geographic areas are still focused on its negative impacts. Thus, the knowledge of the services done by common gorse to the ecosystems is less known. The desirable effects of this plant should be assessed and considered in the design of integrated pest management  strategies. Such research may ultimately lead to better management approaches of invasive weeds and the conservation of native wildlife.

## Data Availability

All relevant information used in the manuscript is available on request.
